# Valorization of bamboo shoot shell waste for the coproduction of fermentable sugars and xylooligosaccharides

**DOI:** 10.3389/fbioe.2022.1006925

**Published:** 2022-09-16

**Authors:** Qiyao Wang, Yan Su, Yang Gu, Chenhuan Lai, Zhe Ling, Qiang Yong

**Affiliations:** ^1^ Jiangsu Co-Innovation Center of Efficient Processing and Utilization of Forest Resources, College of Chemical Engineering, Nanjing Forestry University, Nanjing, China; ^2^ Key Laboratory of Forestry Genetics and Biotechnology (Nanjing Forestry University), Ministry of Education, Nanjing, China

**Keywords:** bamboo shoot shell, enzymatic hydrolysis, hydrothermal pretreatment, glucose, xylooligosaccharides

## Abstract

In this work, hydrothermal pretreatment (autohydrolysis) was coupled with endo-xylanase enzymatic hydrolysis for bamboo shoot shell (BSS) to produce glucose and valuable xylooligosaccharides (XOS) rich in xylobiose (X2) and xylotriose (X3). Results showed that the enzymatic hydrolysis efficiency of pretreated BSS residue reached 88.4% with addition of PEG during the hydrolysis process. To enrich the portions of X2–X3 in XOS, endo-xylanase was used to hydrolyze the XOS in the prehydrolysate, which was obtained at the optimum condition (170°C, 50 min). After enzymatic hydrolysis, the yield of XOS reached 25.6%, which contained 76.7% of X2–X3. Moreover, the prehydrolysate contained a low concentration of fermentation inhibitors (formic acid 0.7 g/L, acetic acid 2.6 g/L, furfural 0.7 g/L). Based on mass balance, 32.1 g of glucose and 6.6 g of XOS (containing 5.1 g of X2-X3) could be produced from 100.0 g of BSS by the coupled technology. These results indicate that BSS could be an economical feedstock for the production of glucose and XOS.

## Introduction

The world needs to focus on sustainable energy development due to the nonrenewable nature of traditional fossil energy. It is urgent to find renewable energy to meet the current energy demand. At the same time, the utilization and development of renewable resources need to become the center of energy policies in many countries. Lignocellulosic resources have the potential to serve as sources of sustainable energy and bio-based materials that can be utilized by biorefineries to alleviate the pressure created by the current energy demand (Huang et al., 2022a; Pei et al., 2022). The main concerns in the biorefinery process are to extract the maximum value from the materials and reduce production costs (Rezania et al., 2020; Huang et al., 2022b). Previous research on biorefineries has mainly focused on agricultural waste (Ma et al., 2021a), while less research has reported the use of food processing waste. For example, bamboo shoot shell (BSS) consists of leaves of bamboo shoots, and the annual output in China exceeds 30 million tons (Ye et al., 2014). In the past, BSS was incinerated and sent to landfill sites rather than being used effectively, resulting in a considerable waste of resources. BSS has a great cost advantage over other lignocellulosic resources used as energy sources (Ma et al., 2022; Yu et al., 2022). As a type of food processing waste, BSS is inexpensive to acquire. Furthermore, direct purchases from factories reduce the high cost of recycling. In addition, the abundant cellulose and hemicellulose in BBS can greatly enhance the commercial value of the biological refinement of BSS. The partial development and application of hemicellulose will create additional value that helps compensate for the economically inefficient production of ethanol from cellulose (Tang et al., 2019). It should be noted that production costs need to be considered when selecting the pretreatment method for BSS, and the economic value of cellulose and hemicellulose after pretreatment should be maintained as much as possible (Ma et al., 2021b).

The idea of coproducing glucose and XOS builds on previous investigations into high-value fractions developed from lignocellulose (Zhang et al., 2022). The idea is that the extra value of XOS obtained from the coproduction process can increase economic efficiency and reduce the cost during ethanol production (Tang et al., 2019). There are increasing numbers of investigations into the coproduction of XOS and glucose, where the efficiency depends on the selection of pretreatment methods (Ma et al., 2021c). Autohydrolysis stands out among many methods as it is inexpensive, simple, and environmentally friendly. Fang et al. (2022) obtained XOS and glucose from birch via autohydrolysis (170°C, 70 min). The efficiency of enzymatic hydrolysis (enzyme dosage 25 FPU/g) reached 89.4% after adding the surfactant Tween 80, and the yield of XOS with a degree of polymerization (DP) 2-6 also reached 46.1%. Zhang X. et al. (2020) obtained glucose and XOS (DP 2-5) from bagasse *via* autohydrolysis with seawater at 175°C for 30 min. After pretreatment, an enzymatic hydrolysis efficiency of 94.7% could be obtained for the pretreated bagasse, with an enzyme dosage of 30 FPU/g and Tween 80. In addition, the XOS (DP 2-5) yield reached 67.1%. The results showed that autohydrolysis could effectively coproduce glucose and XOS. Inhibitors of fermentation are usually generated during autohydrolysis. Furfural and 5-HMF inhibit the growth of fermenting strains (Zaldivar et al., 2015). Dupont and Suarez (2006) reported that when the concentrations of furfural and hydroxymethyl furfural in the pretreatment liquid exceeded 1 g/L, microbial fermentation could be inhibited. Therefore, the application value of the coproduction of glucose and XOS should be reappraised after taking into consideration the existence of fermentation inhibitors (Dai et al., 2021, 2022).

Autohydrolysis is a suitable method to coproduce glucose and XOS. It is a reaction that occurs in the temperature range of 160°C–240°C with an aqueous medium and does not require additional chemical reagents (Zhuang et al., 2016). Under high-temperature and high-pressure conditions, acetyl groups and some glucuronic acid groups are removed from the hemicellulose units in lignocellulose and then combine with water molecules to form acetic acid and glucuronic acid, respectively. The resultant hydrogen ions from the water molecules can form a weakly acidic reaction environment. After autohydrolysis, a small amount of cellulose and most of the xylan in lignocellulose are degraded to form glucooligosaccharides, XOS, glucose, and xylose. In addition, the degraded components can improve the accessibility of cellulose to cellulase. As the autohydrolysis reaction system is a weakly acidic environment, the number of produced fermentation inhibitors is relatively small (Kim et al., 2009; Kumar et al., 2009). Under high-intensity autohydrolysis conditions, the processing of xylan by acetic acid results in shedding of xylan in fragments of different lengths, including XOS and xylose (Zhang et al., 2018; Yu et al., 2022). XOS is sensitive to the intensity of the reaction conditions, and high-intensity conditions result in a decrease of DP for XOS. Therefore, the optimization of time and temperature should be considered for production of XOS during autohydrolysis.

XOS can be considered single-chain sugars containing 2–20 xyloses, which are linked by the *β*-1, 4-glycosidic bonds that are formed from loss of water. XOS is a proven prebiotic that can be easily absorbed by intestinal flora. The improvement of intestinal flora activity will have positive effects on human health, for which *Bifidobacterium* makes a great contribution compared to other intestinal flora (Ghosh et al., 2021). As reported, *Bifidobacterium* is one of the important genera among the microbes in the human gut (Ai et al., 2018; Kim et al., 2020). In addition, X2–X3 are preferentially absorbed by *Bifidobacterium*. Hence, the hydrolysis of the prehydrolysate with endo-xylanase to prepare XOS including more X2–X3 has been proven to be a successful method of enhancing the physiological activity of XOS. Su et al. (2021) used endo-xylanase to improve the component ratio of X2–X3 to XOS, and a greater proportion of X2–X3 was observed in the resultant XOS. After enzymatic hydrolysis by endo-xylanase, the yield of XOS in the prehydrolysate decreased from 30.9 to 25.6%, but the X2–X3 proportion increased from 19.7 to 76.7%. Considering the positive effects of X2 and X3 in XOS on human health, the X2–X3 proportion can be used as one of the evaluation indices for XOS. High-value XOS, which contains more X2 and X3, makes the coproduction of glucose and XOS a competitive option.

In this study, a combination of autohydrolysis and enzymatic hydrolysis was used to prepare glucose and XOS (rich in X2–X3) from BSS. Specifically, the effects of autohydrolysis on enzymatic hydrolysis and the XOS yield of BSS were systematically studied. The cellulase accessibility and the removal rate of xylan were correlated with the enzymatic digestibility of BSS. In addition, the effect of surfactants on enzymatic performance in the hydrolysis of the BSS residue was further investigated. Endo-xylanase hydrolysis was used to increase the proportion of X2–X3 in XOS. It is hoped that this work will provide advanced insights into the coproducing of glucose and high-value XOS from BSS.

## Materials and methods

### Materials

The used BSS was purchased from the Bamboo Processing Factory, which is located in Guilin, Guangxi Province, China. The chemical composition of the BSS was analyzed according to the protocol of the National Renewable Energy Laboratory (Sluiter et al., 2011). The used enzyme of Cellic CTec2 cellulase was supplied by Novozymes NA, Franklinton, United States. The endo-β-1,4-xylanase was supplied by Jiangsu Kangwei Biotechnology Co., Ltd., Yancheng, Jiangsu, China.

### Autohydrolysis of BSS

For autohydrolysis of BSS, 10.0 g of dry BSS (20–60 mesh) and 100 ml distilled water were mixed in the reactor (150 ml). The pretreatment was carried out at 150–190°C for 20–80 min in an oil bath. After pretreatment, the reactor was moved immediately from the oil bath and soaked in a cold-water bath for 4 h. Next, the prehydrolysate in the reaction mixture was separated by filtration. The obtained solid (pretreated BSS) was washed with distilled water until neutralized. Finally, the washed BSS solid and prehydrolysate were stored at 4°C for further analysis.

### Enzymatic hydrolysis of pretreated BSS

The substrate (2%, w/v) was adjusted to pH 4.8 with a citric acid buffer (0.05 M) during enzymatic hydrolysis with a cellulase dosage of 20 FPU/glucan. The enzymatic hydrolysis was performed for 72 h at 150 rpm and 50°C. After enzymatic hydrolysis, the solid and liquid parts were separated using a centrifuge at 8,000 rpm for 5 min. The sugar content in the enzymatic hydrolysate was analyzed using high-performance liquid chromatography (HPLC).

To further improve the enzymatic digestibility of the pretreated BSS, the surfactants of Tween 80 and PEG were used to reduce the negative effect of lignin (Chen et al., 2016; Lai et al., 2018). Specifically, the surfactant (0.075 g/glucan) was incubated in the mixture at 50°C for 30 min before the addition of cellulase.

### Analysis of the accessibility of pretreated BSS

The cellulose accessibility was determined according to work of Inglesby and Zeronia (2002). Specifically, a mixture containing the BSS residue (1%, w/v) and Congo red staining (0, 0.05, 0.1, 0.5, 1.0, 2.0, 3.0, and 4.0 g/L) was shaken at 60°C and 150 rpm for 24 h. Then, cellulase accessibility was calculated by the different absorbance values of the supernate of the mixture at 498 nm. The Langmuir function was used to calculate the accessibility.

### Enzymatic hydrolysis of the prehydrolysate by endo-xylanase

The endo-xylanase activity was determined according to the method established by Bailey et al. (1992). The prehydrolysate obtained at optimum pretreatment conditions (170°C, 50 min) was hydrolyzed by endo-xylanase (3 IU/ml) at 50°C and 150 rpm for 48 h. Aliquots were withdrawn at 4, 8, 12, 24, 36, and 48 h to analyze the content of X2–X3. Once an aliquot was removed, the enzyme reaction was stopped by boiling for 5 min, and the aliquot was kept at 4°C.

### Analysis methods

The HPLC (1260, USA) system containing an Aminex Bop-Rad HPX-87 column was used to analyze the concentration of monosaccharides and inhibitors. H_2_SO_4_ (5 mM) was used as the eluent (0.6 ml/min) during analysis. High-performance anion-exchange chromatography (HPAEC, Dionex 3000) containing a CarboPacPA-200 anion-exchange column was used to analyze the concentration of xylobiose (X2), xylotriose (X3), xylotetraose (X4), xylopentaose (X5), and xylohexaose (X6). NaOH (100 mM) and NaAc (500 mM) containing NaOH (100 mM) were used as eluents at 0.3 ml/min.

The content of XOS was determined according to the xylose difference between the hydrolyzed prehydrolysate [using 8% sulfuric acid (121°C, 60 min)] and the unhydrolyzed prehydrolysate. The enzymatic hydrolysis efficiency, XOS yield, and removal yield of xylan/lignin were calculated according to the following equations:

$$Enzymatic\;hydrolysis\;efficiency=\frac{Glucose\;in\;enzymatic\;hydrolysate\;\left(g\right)}{Glucan\;in\;BSS\;residue\;\left(g\right)\times 1.11}\;\times 100\%.$$ (1)

$$XOS\;yield\;=\frac{Xylose\;\left(sulfuric\;acid\;hydrolyzed\right)\;-Xylose\;\left(unhydrolyzed\right)}{Xylan\;in\;raw\;BSS\;\left(g\right)}\;\times \;0.88\;\times 100\%.$$ (2)

$$Removal\;yield\;of\;xylan/lignin\;=1-\frac{Xylan/lignin\;in\;pretreated\;BSS\;\left(g\right)}{Xylan/lignin\;in\;raw\;BSS\;\left(g\right)}\;\times 100\%.$$ (3)

$$Recovery\;of\;solid/glucan\;=\frac{Solid/glucan\;in\;pretreated\;BSS\;\left(g\right)}{Solid/glucan\;in\;raw\;BSS\;\left(g\right)}\;\times 100\%.$$ (4)

## Results and discussion

### Composition analysis of the BSS residue

For the hydrothermal pretreatment at high temperature and pressure, a weakly acidic environment can be formed to degrade the xylan in BSS into XOS and xylose (Kim et al., 2009; Kumar et al., 2009). Hence, the compositions of the pretreated BSS were determined and shown in Table 1. It can be seen that the reaction temperature and time significantly affected the composition of the pretreated BSS. For example, the solid yield of recovery decreased from 76.3 to 58.2%, and the removal yield of xylan sharply increased from 34.5 to 92.9% with the temperature increased from 150 to 190°C. In contrast, the recovery yield of glucan slightly decreased from 87.3 to 84.0%, and the removal yield of lignin increased from 18.8 to 23.2% under the same pretreatment conditions. The dramatic decrease of xylan recovery indicates that autohydrolysis has a promising ability to remove xylan. However, autohydrolysis showed a limited contribution in removing lignin. Under the high-energy environment, the electrons of water molecules will be able to escape from the system of water molecules, which will cause the water molecules to be ionized, and this trend of ionization will become stronger with the increase of the pretreatment intensity (Yue et al., 2021). Xylan is selectively ionized by ionization of water during autohydrolysis (Lu et al., 2016). With an increase in temperature, the degree of ionization of water can be increased, which is beneficial for removal of xylan. Meanwhile, the high recovery yield of glucan indicates that autohydrolysis has the potential to produce cellulose from BSS for further bioconversion (Lian et al., 2022).

**TABLE 1:** Compositions of the BSS residue after autohydrolysis.

Temperature (°C)	Time (min)	Composition (%)			Removal yield (%)		Recovery yield (%)		Γmax/DR28
		Glucan	Xylan	Lignin	Solid	Glucan	Xylan	Lignin	(mg/g)
BSS	\	38.2 ± 1.0	25.7 ± 0.1	25.6 ± 0.2	\	\	\	\	264.2
150	60	42.1 ± 0.1	21.4 ± 0.2	26.3 ± 0.2	76.3 ± 0.1	87.3 ± 0.1	34.5 ± 0.0	18.4 ± 0.3	401.9
160		44.5 ± 0.2	19.7 ± 0.5	27.8 ± 0.0	74.6 ± 0.1	86.9 ± 0.1	43.2 ± 0.0	18.8 ± 0.2	439.7
170		49.3 ± 0.2	9.6 ± 0.5	30.3 ± 0.0	66.9 ± 0.0	86.1 ± 0.0	75.2 ± 0.1	20.7 ± 0.0	457.8
180		53.5 ± 0.7	5.59 ± 0.6	32.7 ± 0.4	60.8 ± 0.2	85.1 ± 0.0	87.1 ± 0.2	22.1 ± 0.3	405.2
190		55.2 ± 0.9	3.2 ± 0.5	33.2 ± 0.0	58.2 ± 0.0	84.0 ± 0.1	92.9 ± 0.1	23.2 ± 0.5	361.8
170	20	45.3 ± 0.5	17.9 ± 0.8	27.6 ± 1.2	73.8 ± 0.1	87.3 ± 0.4	48.9 ± 0.0	15.8 ± 0.2	425.4
	30	46.1 ± 0.0	16.6 ± 0.7	28.7 ± 0.8	72.4 ± 0.0	87.2 ± 0.2	53.7 ± 0.1	16.2 ± 0.2	403.4
	40	47.8 ± 0.3	14.2 ± 0.4	29.8 ± 0.3	69.5 ± 0.5	87.0 ± 0.0	61.9 ± 0.0	18.3 ± 0.0	450.3
	50	49.0 ± 0.7	12.0 ± 0.4	30.6 ± 0.1	67.2 ± 0.7	86.5 ± 0.1	68.8 ± 0.3	20.4 ± 0.4	509.9
	60	49.3 ± 0.2	9.6 ± 0.5	30.3 ± 0.0	66.9 ± 0.0	86.1 ± 0.2	75.2 ± 1.1	20.7 ± 0.1	457.8
	70	51.2 ± 0.2	6.9 ± 0.2	32.0 ± 0.1	63.8 ± 0.1	85.5 ± 0.1	83.0 ± 0.2	21.3 ± 0.2	442.7
	80	51.5 ± 0.3	6.8 ± 0.1	32.5 ± 0.2	63.3 ± 0.1	85.2 ± 0.1	83.4 ± 0.0	21.7 ± 0.1	441.0

To further optimize pretreatment conditions for BSS, the pretreatment time was increased from 20 min to 80 min. The changes in the compositions of BSS are shown in Table 1. It can be seen that the recovery yield of the solid residue decreased from 73.8 to 63.3%, and the xylan removal yield increased from 48.9 to 83.4%. It seems, therefore, that glucan degradation is not sensitive to a prolonged reaction time. The recovery yield of glucan decreased only slightly from 87.3 to 85.2%, and the removal of lignin increased from 15.8 to 21.7% when the pretreatment time was increased from 20 to 80 min. These results indicate that the pretreatment duration should also be considered one of the factors affecting the removal of xylan and lignin. Hence, prolonging the pretreatment time also improves the removal of xylan and lignin from BSS during autohydrolysis.

Based on the aforementioned results, it can be seen that autohydrolysis at 170°C for 50 min showed best performance for removing xylan and lignin from BSS. The removal yield of xylan reached 68.8%, which may be of great significance for improving the enzymatic hydrolysis of cellulose into glucose (Huang et al., 2018). The removal of lignin reached 20.4%, which can also be considered a positive factor for improving enzymatic efficiency (Ma et al., 2020; Paz-Cedeno et al., 2021). However, the non-production adsorption interaction between residual lignin and cellulase will reduce the efficiency of enzymatic hydrolysis (Yu et al., 2022). Hence, an approach to intervene in the interaction should be carried out to improve the enzymatic efficiency (Li et al., 2022).

### Composition analysis of the BSS prehydrolysate

Autohydrolysis results in degradation of the xylan from the lignocellulose into prehydrolysate, including subfractions of glucan and lignin. During the autohydrolysis process, xylan can be broken down into XOS and xylose. In addition, fermentation inhibitors formed from the monomer sugar subsequently undergo a series of reactions (Sipos et al., 2009). Hence, the analysis of the components in the prehydrolysate was performed to understand the amount of dissolved degradation products from BSS during the autohydrolysis process.

Based on the aforementioned results in the “Composition analysis of BSS residue” section, it can be seen that the pretreatment temperature and time affected the removal yield of xylan. The ionization of water results in the degradation of xylan into the liquid phase as XOS. Table 2 shows that the quantity of XOS increased from 4.1 g/100 to 8.2 g/100 g (150–170°C, 60 min), but it decreased from 8.2 g/100 to 4.9 g/100 g when the temperature continued to increase (170–190°C, 60 min). Thhis phenomenon can be explained by the fact that *β*-1, 4 glycosidic bonds in XOS were broken by the increased acidic environment owing to the increased pretreatment temperature. Wang et al. (2019) also found that the content of XOS began to decline at temperatures over 170°C during autohydrolysis for eucalyptus. To obtain a higher yield of XOS, it was necessary to optimize the reaction time. In Table 2, it can also be seen that the content of XOS increased with the increase in the pretreatment time from 20 to 80 min at 170°C. Fang et al. (2022) also found that prolonging the reaction time of autohydrolysis promoted the production of XOS from birch.

**TABLE 2:** Sugars and byproducts in prehydrolysates of BSS after autohydrolysis.

Temperature (°C)	Time (min)	Fermentation inhibitor (g/L)				Sugar in prehydrolysate (g/100 g)			
		Formic acid	Acetic acid	5-HMF	Furfural	Glucose	Gluco-oligosaccharide	Xylose	XOS
150	60	0.5 ± 0.0	1.6 ± 0.1	0.0 ± 0.0	0.0 ± 0.0	0.4 ± 0.0	0.8 ± 0.0	0.2 ± 0.0	4.1 ± 0.0
160		0.5 ± 0.1	2.0 ± 0.0	0.0 ± 0.0	0.0 ± 0.0	0.4 ± 0.0	0.9 ± 0.0	0.3 ± 0.0	6.5 ± 0.1
170		0.7 ± 0.0	2.9 ± 0.0	0.0 ± 0.0	1.0 ± 0.1	0.5 ± 0.1	0.9 ± 0.1	1.4 ± 0.3	8.2 ± 0.3
180		0.9 ± 0.0	4.5 ± 0.0	0.0 ± 0.0	3.6 ± 0.2	0.6 ± 0.0	1.1 ± 0.0	3.1 ± 0.4	6.3 ± 0.0
190		0.9 ± 0.0	5.3 ± 0.0	0.2 ± 0.0	5.3 ± 0.1	1.4 ± 0.3	0.6 ± 0.0	3.2 ± 0.2	4.9 ± 0.2
170	20	0.4 ± 0.0	1.5 ± 0.2	0.0 ± 0.0	0.0 ± 0.0	0.4 ± 0.0	0.6 ± 0.1	0.4 ± 0.1	4.6 ± 0.4
	30	0.5 ± 0.0	2.0 ± 0.0	0.0 ± 0.0	0.0 ± 0.0	0.4 ± 0.0	0.7 ± 0.0	0.5 ± 0.0	6.0 ± 0.2
	40	0.6 ± 0.0	2.5 ± 0.0	0.0 ± 0.0	0.3 ± 0.0	0.5 ± 0.0	0.8 ± 0.0	0.7 ± 0.0	7.9 ± 0.0
	50	0.7 ± 0.0	2.6 ± 0.1	0.0 ± 0.0	0.7 ± 0.1	0.5 ± 0.1	0.9 ± 0.0	1.1 ± 0.2	8.0 ± 0.1
	60	0.7 ± 0.0	2.9 ± 0.0	0.0 ± 0.0	1.0 ± 0.0	0.5 ± 0.1	0.9 ± 0.1	1.4 ± 0.0	8.2 ± 0.1
	70	0.9 ± 0.0	3.2 ± 0.0	0.0 ± 0.0	1.2 ± 0.0	0.6 ± 0.0	0.9 ± 0.1	1.8 ± 0.1	8.4 ± 0.1
	80	0.9 ± 0.1	3.5 ± 0.0	0.0 ± 0.0	1.5 ± 0.3	0.6 ± 0.1	1.0 ± 0.0	2.1 ± 0.2	8.8 ± 0.2

Fermentation inhibitors are also common byproducts during autohydrolysis. As shown in Table 2, both an increase in temperature and prolongation of reaction time resulted in an improvement of the content of formic acid and acetic acid, while it was notable that 5-HMF only appeared at 190°C. The concentration of XOS in the prehydrolysate should be considered when the aim is the coproduction of XOS and glucose. Based on the entire process, it can be seen that autohydrolysis at 170°C for 40 min was the optimum condition to produce XOS with lower furfural and 5-HMF from BSS.

### Enzymatic hydrolysis of the BSS residue

**FIGURE 1:**
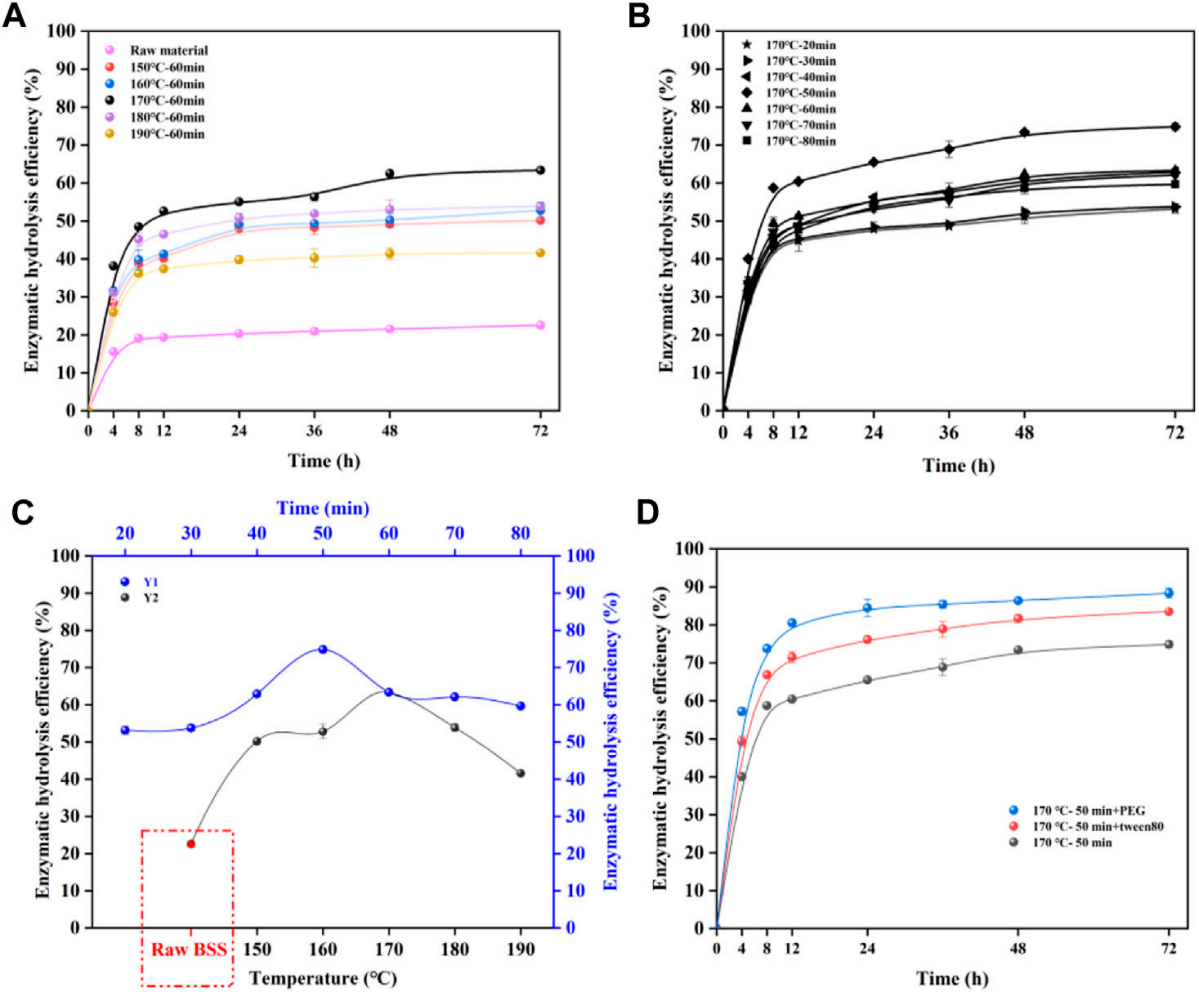
Effects of pretreatment temperature **(A)**; duration **(B)**; and surfactant **(D)** on enzymatic hydrolysis; enzymatic hydrolysis efficiency of 72 h **(C)**, (Y1) pretreatment time, (Y2) pretreatment temperature.

Enzymatic hydrolysis efficiency is an extremely important evaluation criterion for the utilization of cellulose in wood (Li et al., 2018). Figures 1A,B display the enzymatic hydrolysis yields of pretreated BSS with cellulase (20 FPU/g glucan) for 72 h. It can be seen that the hydrolysis efficiency of the pretreated BSS was significantly greater than that of raw BSS. The change in the efficiency may be explained by the fact that the hemicellulose was removed to form more pores, which resulted in more active sites for enzyme binding from residual cellulose (Yu et al., 2022). Zhang et al. (2013) also that found increasing the accessibility of cellulose during pretreatment was due to xylan removal. Hence, it can be speculated that autohydrolysis can substantially improve the enzymatic digestibility of BSS.

Figure 1C shows the enzymatic hydrolysis efficiency at 72 h. The optimized hydrolysis results of pretreated BSS can be clearly seen with a small error for parallel samples, indicating the accuracy of enzymatic hydrolysis efficiency. In Figure 1C (Y2), the efficiency of enzymatic hydrolysis increased from 50.2 to 63.4% when the pretreatment increased from 150°C to 170°C at 60 min. In addition, the enzymatic hydrolysis efficiency of pretreated BSS (170°C, 60 min) was 63.4%, which was clearly higher than that of raw BSS (22.5%). The increased enzymatic hydrolysis efficiency can be attributed to the increased removal of hemicellulose, while the efficiency decreased rapidly from 63.4 to 41.6% when pretreatment increased from 170°C to 190°C at 60 min. This could be because the increasingly ineffective adsorption of the enzyme by the lignin prevented the enzyme from fully binding to the substrate, thereby decreasing the enzymatic hydrolysis efficiency. Tang et al. (2021) found that the removal of hemicellulose increased the ineffective adsorption capacity of lignin for enzymes. A natural surfactant (humic acid, HA) was successfully used to prevent the ineffective adsorption of enzymes, and this improved the enzymatic hydrolysis efficiency. The trend in enzymatic hydrolysis efficiency reflects the fact that the performance of the enzymatic hydrolysis of pretreated BSS has a strong relationship with temperature. It can be clearly seen that the use of a temperature of 170°C conferred a significant advantage by improving the performance of the enzymatic hydrolysis of BSS after autohydrolysis. As shown in Figure 1C (Y1), the optimum reaction time can be found at 170°C. It was found that the substrate (170°C, 50 min) achieved the best enzymatic hydrolysis efficiency (74.8%). Figure 1C showed that the hydrolysis efficiency of the BSS residue (170°C, 50 min) was significantly higher than that of raw BSS, which was 74.8%.

To make a further improvement to the enzymatic hydrolysis efficiency, nonionic surfactants (PEG, Tween 80) were added to the reaction mixture to reduce the ineffective adsorption of lignin on cellulase (Figure 1D). The results revealed that the enzymatic hydrolysis efficiency increased significantly after the addition of PEG/Tween 80 to the enzymatic system of pretreated BSS (170°C, 50 min), which could achieve a yield of 86.6% (Tween 80) and 88.4% (PEG). It has been reported that the addition of surfactants could reduce the adsorption ability of lignin for enzymes, resulting in freer cellulase in the system for hydrolysis of the cellulose substrate (Huang et al., 2022c). Overall, the enzymatic hydrolysis efficiency of BSS improved substantially after autohydrolysis, and the optimization of temperature and duration further improved it. The addition of surfactants can also improve its enzymatic digestibility efficiency.

### The relationships between the removal of xylan, cellulose accessibility, and the efficiency of the enzymatic hydrolysis of pretreated BSS

**FIGURE 2:**
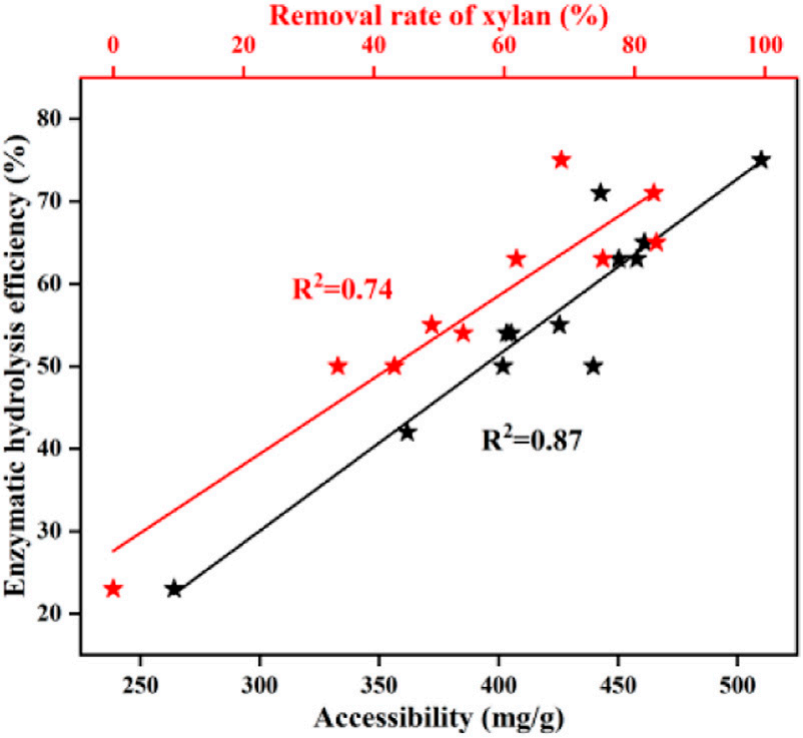
Relationships between the structural properties and the efficiency of the enzymatic hydrolysis of pretreated residues.

The results of enzymatic hydrolysis had a close connection to the degree of xylan removal (Kruyeniski et al., 2019). As shown in Figure 2, the enzymatic efficiency of pretreated BSS increased with increasing xylan removal. There was a correlation between the performance of the enzymatic hydrolysis and the removal of xylan (*R* ^2^ = 0.74). Huang et al. (2018) also found a similar relationship between the amount of xylan removed and the performance of the enzymatic hydrolysis of pretreated bamboo residues. This was due to the fact that the surface area of the residual solid cellulose increased, caused by the removal of xylan, which provided more active sites for cellulase. In addition, both Hu et al. (2012) and Zhang et al. (2013) found that the removal of xylan will contribute to increasing the accessibility of cellulose to cellulases. Autohydrolysis displays beneficial effects on the removal of xylan and thereby improves the performance of enzymatic hydrolysis.

The accessibility of cellulose reflects the degree of cellulase and substrate adsorption (Lai et al., 2018). The cellulase accessibility was determined by Congo red and is shown in Table 1. It was found that untreated BSS yielded 264.2 mg/g of an accessible substrate. As expected, the accessibility was greatly enhanced after autohydrolysis, reaching a maximum of 509.9 mg/g (170°C, 50 min). The accessibility of cellulose increased from 401.9 mg/g to 457.8 mg/g (150–170°C, 60 min) and then declined from 457.8 mg/g to 361.8 mg/g (170–190°C, 60 min). After the optimal pretreatment time (170°C, 20–80 min), the accessibility also initially showed an increase and then it decreased, with the best result of 509.9 mg/g (170°C, 50 min). The pattern of change in accessibility is very similar to that of the change in the efficiency of enzymatic hydrolysis. The relationship (*R* ^2^ = 0.87) between accessibility and enzymatic hydrolysis efficiency is linear, which is consistent with the work of Huang et al. (2019). Autohydrolysis breaks the lignocellulosic structural bonds, leading to hemicellulose being removed and lignin redistribution, improving cellulose accessibility resulting from increasing the surface area. As a result, the performance of enzymatic hydrolysis is increased (Kellock et al., 2019; Zhang Q. et al., 2020; Li et al., 2020). Generally, the increased accessibility of cellulase has a positive effect on the efficiency of the enzymatic hydrolysis of BSS with autohydrolysis.

### Enzymatic hydrolysis of the prehydrolysate for X2–X3 production

Compared to XOS with a high average DP, it has been suggested that X2–X3 are better at making use of *Bifidobacterium* (Chen et al., 2016). XOS with a higher quantity of X2–X3 will increase the proliferative activity of *Bifidobacterium*. Ai et al. (2018) found that the XOS group with a high X2–X3 proportion was more selective for beneficial bacteria than the higher XOS group (DP > 3). To improve the activity of XOS, it is recommended to increase the X2–X3 proportion of XOS. In this work, the endo-xylanase was used to hydrolyze the prehydrolysate of BSS to increase the proportion of X2–X3 (Su et al., 2021).

**FIGURE 3:**
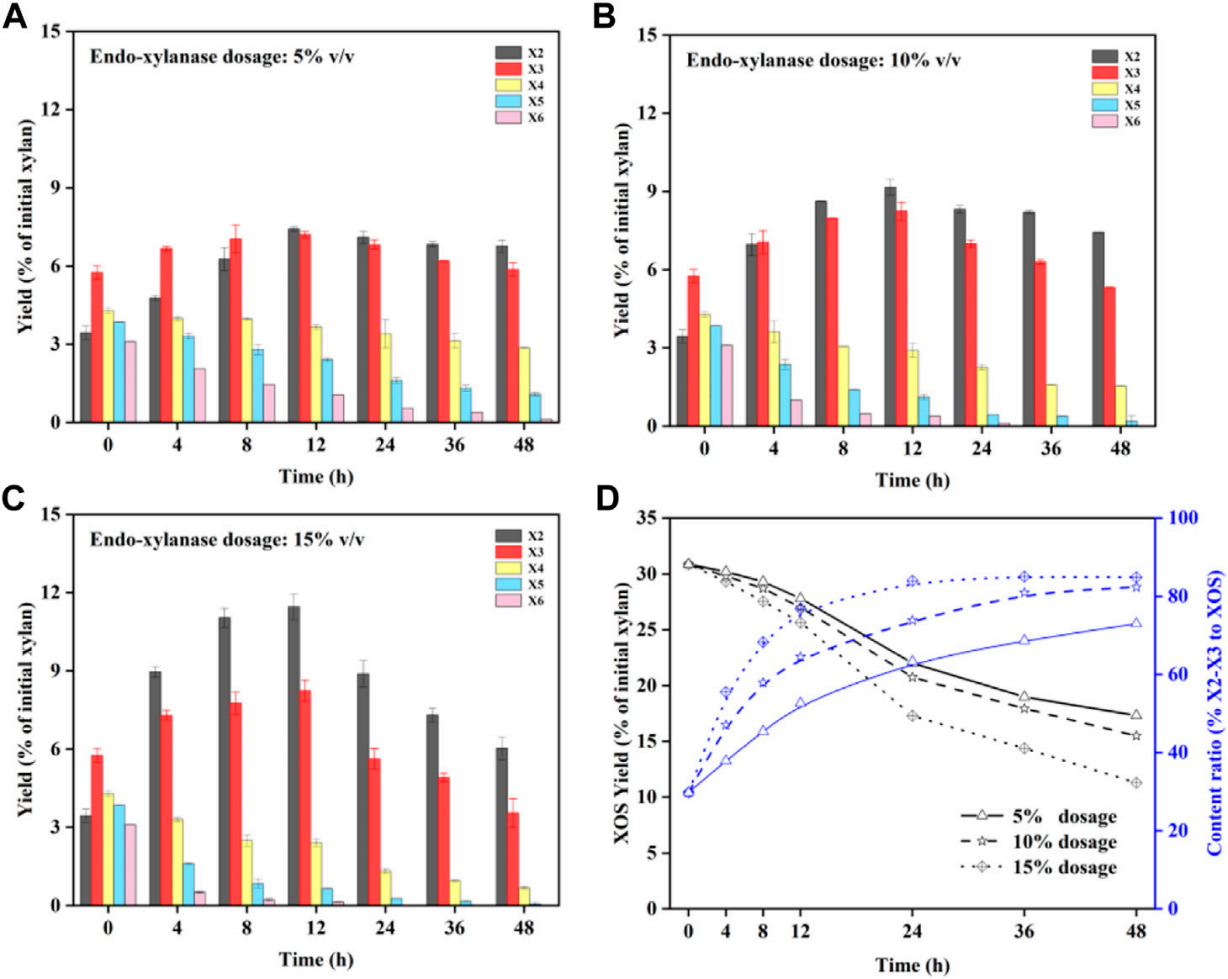
Distribution of X2–X6 during enzymatic hydrolysis with different enzyme dosages: 5% **(A)**; 10% **(B)**; 15% **(C)**; concentration of X2–X3 and X2–X3 proportion of XOS **(D)**.

The results of endo-xylanase hydrolysis are displayed in Figure 3. Figures 3A–C display the quantity of X2–X6 in the prehydrolysate after enzymatic hydrolysis with the endo-xylanase dosage of 5–15% (v/v). It can be seen that X2–X3 content could be clearly increased after enzymatic hydrolysis for an extended time. In Figure 3A, the amounts of X4–X6 were decreased during the enzymatic hydrolysis time, with a degradation rate of X6 > X5 > X4. It has been confirmed that XOS with high DP has more enzyme binding sites for enzymatic hydrolysis in a higher priority at a similar initial content (Su et al., 2021). Meanwhile, the content of X2–X3 showed a slight increasing trend during 0–12 h. This can be attributed to the degradation of X4–X6 into X2–X3 by endo-xylanase. Based on the results, it can be seen that the accumulation of X2–X3 was originated from the degradation of XOS (DP > 3) and that a higher dosage of the enzyme will enhance the efficiency of degradation (Figure 3C). Therefore, the yield of XOS should be taken into consideration when the maximum ratio of X2–X3 is selected. The change of the X2–X3 ratio to XOS and the XOS yield under different enzyme dosages is shown in Figure 3D. It can be seen that XOS with a 15% enzyme dosage decreased faster than enzyme dosages of 5 and 10%, in which high DP will be hydrolyzed preferentially. Although the X2–X3 ratio of XOS is much higher with prolonged time, the total yield of XOS decreases. Hence, it can be speculated that the conditions of 15% enzyme dosage and 12 h reaction time are beneficial for producing the high-value XOS. Compared to the yield of XOS with 30.9% for the prehydrolysate, a yield of XOS with 25.6% was obtained after it was hydrolyzed by the enzyme, while the X2–X3 proportion in XOS increased to 76.7%, achieving an improvement of 46.9% compared to that in initial XOS. In addition, the XOS satisfies the commercial product standard of 70% purity for autohydrolysis coupled with endo-xylanase hydrolysis (Vázquez et al., 2000).

### Mass balance

**FIGURE 4:**
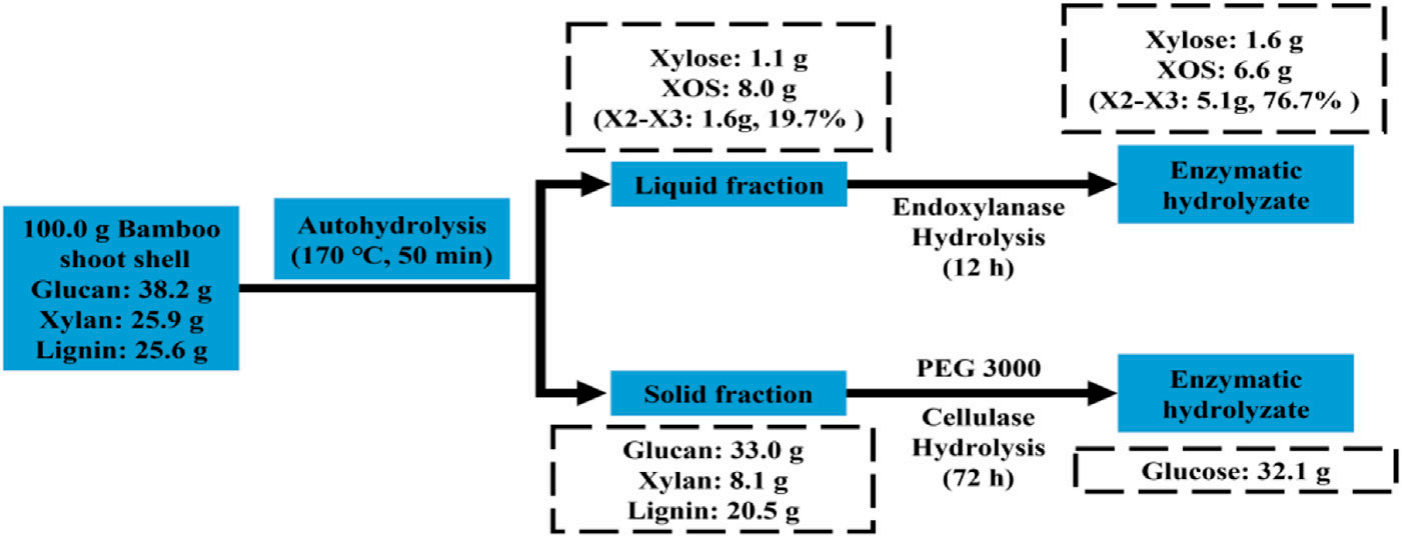
Mass balance of BSS during the established process.

This work demonstrates an efficient and profitable means of coproducing glucose and valuable XOS using a combination of autohydrolysis and enzymatic hydrolysis. As shown in Figure 4, 67.2 g of the residue could be recovered after the autohydrolysis of 100.0 g of BSS, which contained 33.0 g of glucan, 8.1 g of xylan, and 20.5 g of lignin. A total of 32.1 g of glucose was obtained from 67.2 g of pretreated BSS by cellulase hydrolysis. In addition, 1.1 g of xylose and 8.0 g of XOS dissolved in the prehydrolysate, which were degradation products of xylan. The prehydrolysate hydrolyzed by endo-xylanase could produce 1.6 g of xylose and 6.6 g of XOS, which contains a high proportion (76.7%) of X2–X3.

### Analysis of competitive advantages in the coproduction of glucose and XOS from BSS

Autohydrolysis is pretreatment technology that is less corrosive to the equipment, which can improve the service life of the equipment and increase the safety of the production environment (Scapini et al., 2021; Huang et al., 2022d). Compared to other pretreatments, the obtained hydrolyzate possesses fewer fermentation inhibitors by using autohydrolysis. For example, 5 g/L furfural and 3.1 g/L furfural were found in the prehydrolysate from seawater pretreatment (Zhang X. et al., 2020) and synergistic hydrothermal-deep eutectic solvent (DES) pretreatment (Ma et al., 2021b), respectively. It has been reported that 1 g/L furfural can significantly affect the growth of microorganisms and that 5 g/L furfural seriously damages the growth of microorganisms (Zaldivar et al., 2015). In this study, only 0.7 g/L furfural was detected in the XOS. Therefore, the cost of separating the furfural from the XOS was reduced. In addition, the X2–X3 proportion in XOS was 76.7% after enzymatic hydrolysis with endo-xylanase. X2–X3 were the main valuable prebiotics in the XOS (Ai et al., 2018; Kim et al., 2020). Hence, applying this low-intensity method to coproduce glucose and high-value XOS provides a new possibility for the biorefinery of BSS.

## Conclusion

A green and efficient method of coproducing glucose and value-added XOS from BSS has been proposed. The enzymatic efficiency reached 88.4% with the addition of PEG (170°C, 50 min). Under these conditions, the yield of XOS reached a maximum of 25.6%, and the X2–X3 proportion reached 76.7% after endo-xylanase hydrolyzed the prehydrolysate. In addition, the concentration of furfural in the prehydrolysate was only 0.7 g/L. Overall, autohydrolysis coupled with enzymatic hydrolysis can be used to coproduce high-value XOS and glucose from BSS as a suitable raw material for biorefinery.

## Data Availability

The raw data supporting the conclusion of this article will be made available by the authors, without undue reservation.
